# Risk factors associated with malaria outbreak in Laelay Adyabo district northern Ethiopia, 2017: case-control study design

**DOI:** 10.1186/s12889-019-6798-x

**Published:** 2019-05-02

**Authors:** Afewerki Tesfahunegn, Gebretsadik Berhe, Equbay Gebregziabher

**Affiliations:** 1 College of Health Sciences, Mekelle University, Mekelle, Ethiopia; 2 Tigray Health Research Institute, Mekelle, Ethiopia

**Keywords:** Case control, Malaria, Malaria outbreak, Outbreak, Laelay-Adyabo District

## Abstract

**Background:**

Globally in 2015 about 214 million malaria cases and 438,000 deaths were reported with 75% were from Sub-Saharan Africa. Malaria transmission in Ethiopia is unstable, and outbreaks are considered public health emergencies. Understanding the trigger for outbreaks in low-transmission areas can help facilitate malaria elimination. On July 8th malaria outbreak was reported from Laelay Adyabo district. The objective was to investigate the magnitude and associated factors with malaria outbreak.

**Methods:**

We defined a case as confirmed malaria using microscopy or a rapid diagnostic test for *Plasmodium* parasites in a resident of Laelay-Adyabo District from July 9–28, 2017. We identified cases by reviewing health facility records and conducted a case-control study using randomly-selected cases from a line list, and two neighborhood controls per case. A pretested semi-structured questionnaire adapted from WHO malaria guidelines was used to collect data from case-patients and controls. We calculated crude (COR) and adjusted (AOR) odds ratios to identify factors associated with malaria.

**Result:**

A total of 145 confirmed malaria cases (57.9% males) were identified with village attack rate (AR) of 12.1/1000. The AR was higher among males than females (14.1 verses 10.1/1000), children aged 5–14 years (12.9/1000), and in Zelazle *Kebelle* (13.6/1000 population). Wearing protective clothing (AOR = 0.27, 95% CI 0.11–0.66), having good knowledge of malaria transmission (AOR = 0.25, 95% CI 0.08–0.75), having waste collection material at home (AOR = 0.25 95% CI 0.11–0.61), availability of mosquito breeding sites around home (AOR = 9.08, 95% CI 3.6–22.93), and staying outdoor overnight (AOR = 3.7, 95% CI 1.44–9.56) were independently associated with malaria.

**Conclusion:**

The overall attack rate for malaria during this outbreak was high affecting > 1% of the population. Wearing protective clothing at night, knowing about malaria transmission, having mosquito breeding sites around the home, staying outdoors overnight, and having waste collection material in their house were predictors of the infection. Laelay Adyabo district health office should provide health education on malaria transmission and prevention measures and how to clear mosquito breeding sites.

**Electronic supplementary material:**

The online version of this article (10.1186/s12889-019-6798-x) contains supplementary material, which is available to authorized users.

## Background

Malaria is a parasitic infection caused by plasmodium species. Globally about 214 million malaria cases and 438,000 deaths occurred during 2015. Approximately 75% of cases and deaths were from Sub-Saharan Africa; Where approximately 60% of the population at risk [1, 2].

In Ethiopia, malaria transmission is unstable; however, approximately 68% of the populations still live in malaria-endemic areas. Ethiopia is aggressively working towards malaria elimination by 2030 [3–7]. Known risk factors for malaria include low utilization of Insecticidal Treated bed Nets (ITNs), low utilization of Indoor Residual Spray (IRS), availability of multiple mosquito breeding sites or stagnant water sites near the home, and staying outdoors overnight [8–11]. Malaria outbreaks can occur among persons living near irrigation sites and dam areas, persons who keep livestock, live in near river, staying inside home at night, sleeping under ITNs, and spraying of IRS are protective [12–15].

Ethiopian ministry of health on 2015 targets to maintain zero malaria deaths and reduce malaria cases and admission by 67 and 48% respectively [16, 17]. Ethiopia applied interventions like early diagnosis and treatment, and use of vector control methods (indoor residual spray with insecticide and ITNs) to control malaria in the last 25 years. These interventions are highly effective in reducing both the transmission and exposure to infectious mosquito bites and also the concomitant burden of malaria disease. However, ITN ownership and usage levels are still both below target levels. According to 2015 malaria indicator survey, about 64% of households in malaria-endemic areas owned at least one ITN [18–20].

Laelay Adyabo district received report of unexpectedly high number of malaria cases occurring since July 8th, 2017. This was unusual, as no malaria outbreak had happened in the last 10 years in the district [7]. Therefore we conducted a study.

Understanding the reasons for outbreak occurrence in low transmission areas enable to provide early case management, identify factors that maintain the disease, and design more effective prevention and control measures to facilitate malaria elimination strategy by 2030. Therefore, the objective of this study was to investigate the magnitude and risk factors associated with the malaria outbreak in this low-transmission area.

## Methods

### Study setting and period

This study was conducted in Laelay Adyabo district northwestern Tigray region, Ethiopia on July 9–28, 2017. The district has five health centers and 17 health posts. The district was 1924 m above sea level and they had activities like IRS, ITNs distribution to the community in the last 20 years [7]. The outbreak happened in two of the district *Kebelles* (Tsehayo and Zelazle *Kebelle* (total population: 12,000).

### Study design

We did descriptive analysis and conducted a case-control study to identify risk factors associated with malaria.

### Study participant and variables

Confirmed cases were defined as malaria confirmed by microscopy or Rapid Diagnostic Test (RDT) in an individual currently living in the study area. Suspected cases included any person with fever or fever with headache, back pain, chills, rigor, sweating, muscle pain, nausea and vomiting diagnosed clinically as malaria. Controls were healthy neighbors of cases who did not have malaria by RDT during the outbreak period.

We used a standardized questionnaire from literatures to collect data from cases and controls. The questionnaire was administered by trained interviewers to collect data on patient age, sex, residence, family size, insecticide-treated bed net (ITNs) usage, history of indoor residual spraying (IRS) at home, river within 2 km, overnight sleeping location (outdoor or indoor), presence of mosquito breeding sites at home, and knowledge of respondents about malaria transmission (Additional file 1). We also abstracted data from clinical patient records.

### Sample size and sampling technique

Sample size was calculated by assuming an odds ratio of 0.3 for ‘sleeping under ITNs, control exposure of 87.4%, and case to control ratio of 1:2 for a total sample size of 170. With a 10% non-response rate, our sample size was 186, with 62 cases and 124 controls [11]. We selected cases randomly from our line list, and identified two neighborhood controls per case.

### Data processing and analysis

Data were entered to EPI INFO 7.0 after cleaning and coding. Data were analyzed using SPSS 20.0. Descriptive analyses were conducted frequency with percentage for categorical variables and median and Inter Quartile Range (IQR) were computed for continues variables and normality test was checked graphically. Attack rate were computed based on age, sex, and residence of patients. Both bivariate and multivariable logistic regression was performed to identify factors associated with malaria outbreak.

We checked for multi-Collinearity using Variance Inflation Factor (VIF); values < 10 were included in the model. The model was fitted by Hosmer and Lemeshow’s goodness-of-fit. Data were presented using adjusted odds ratio (AOR) with the 95% confidence interval.

### Operational definition

Malaria outbreak: increment of malaria cases in a specific week comparing to the doubling last year of the same weeks.

Wearing Protective cloth: peoples who wore long cloth to protect their leg and hand during night.

Waste collection material: peoples who have waste collection material in around their house.

Good knowledge: peoples who scored above mean of knowledge questions otherwise poor.

Mosquito breeding site: peoples who have stagnant water, availability of mosquito breeding material, and availability of dungs and tick grass in their dwelling house.

Outdoor overnight: peoples who stay outdoor more than 6 h during the night time.

### Ethical consideration

Ethical clearance was obtained from Mekelle University College of Health Science institutional review board. Written informed consent and assent were obtained from participant/caregivers.

## Result

### Descriptive result

**Fig. 1:**
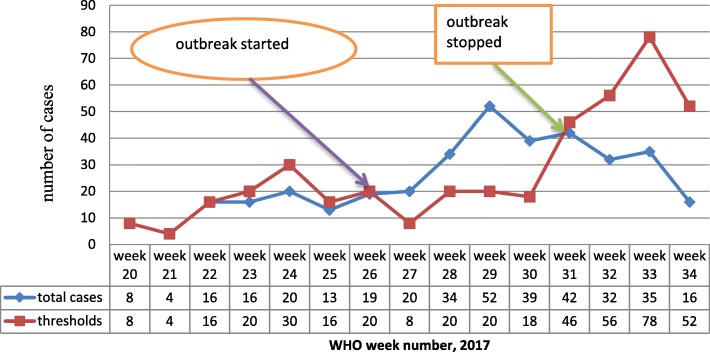
Malaria outbreak line graph in Laelay Adyabo district northern Ethiopia, 2017

A total of 145 confirmed cases of malaria were identified over the outbreak period during WHO weeks 27 to 30 from Tsehayo and Zelazle *Kebelle* (Fig. 1). The village Attack Rate (AR) during the outbreak was 12.1 cases per 1000.

Among the 145 malaria patients, 84 (57.9%) were male. Sex-specific attack rates were 14.1 (males) and 10.1 (females) per 1000 population. Median age of patients was 16 years (IQR: 8–25 years) and approximately half were in the age group of 15–59 years (Table 1).

**Table 1:** Age specific attack rate of malaria outbreak in Laelay Adyabo district northern Ethiopia 2017 (*n* = 145)

Age ranges	No (%)	Age specific attack rate per 1000 population
< 5 years	22 (15.2)	12.8
5–14 year	45 (31)	12.9
≥15 years	78 (53.8)	12.4
Total	145 (100)	12.1

**Fig. 2:**
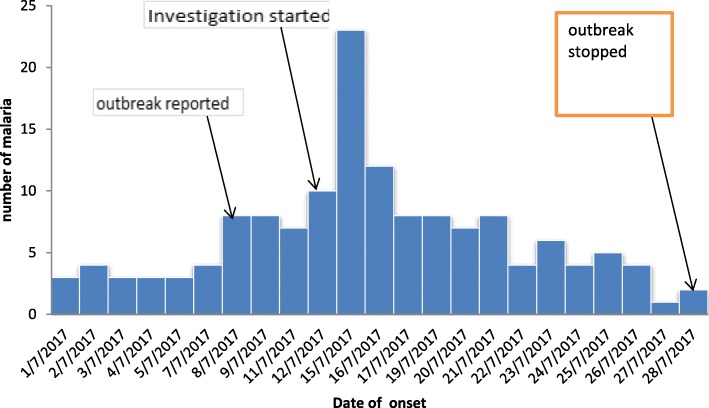
Malaria outbreak Epi curve in Laelay Adyabo district northern Ethiopia, 2017

The date of onset for most of the cases 23 (15.9%) were on July 15, 2017 and the epidemic curve demonstrated a propagated outbreak (Fig. 2).

**Fig. 3:**
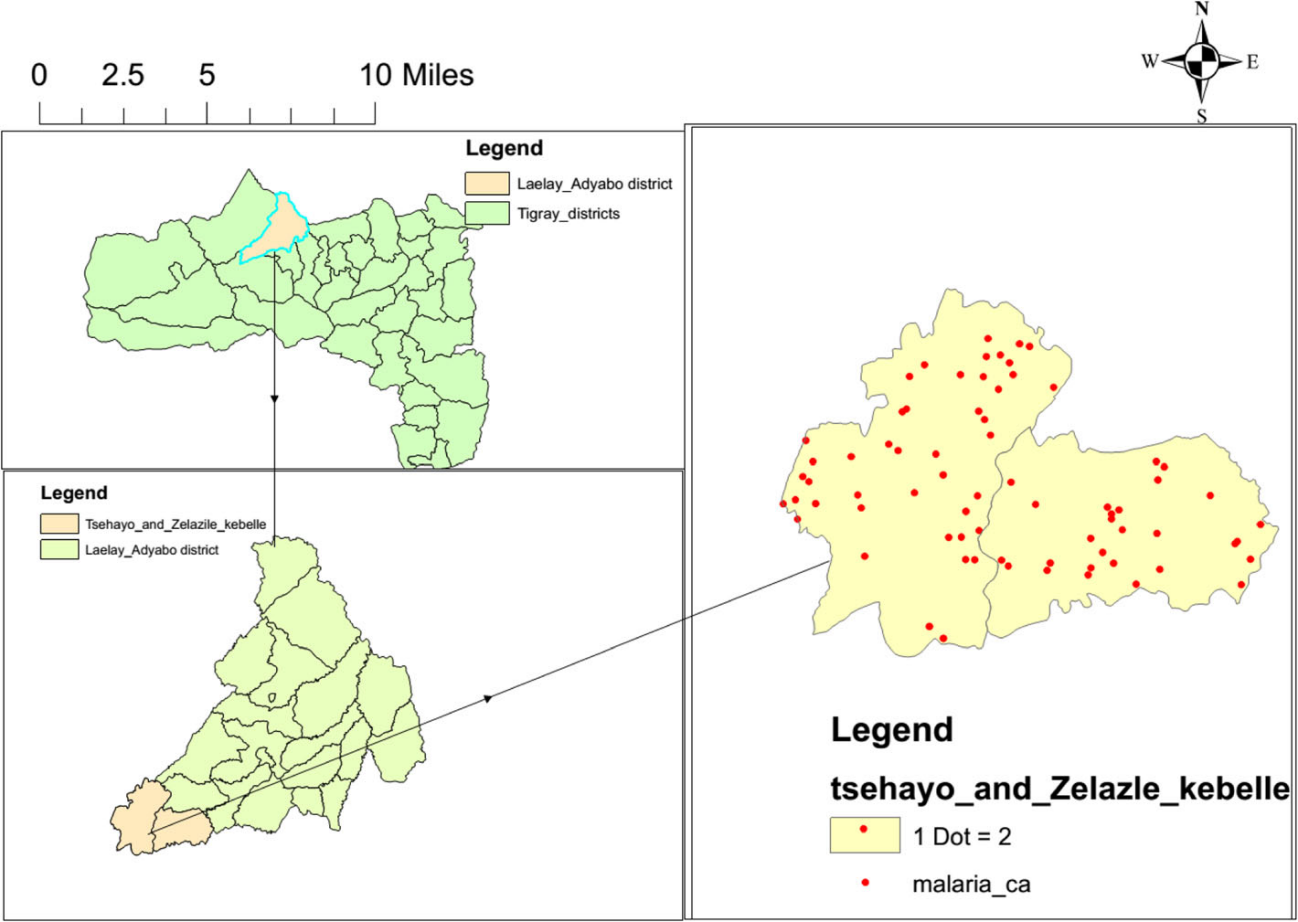
Malaria outbreak affected *Kebelles* spot map in Laelay Adyabo district northern Ethiopia, 2017

Eighty-nine (61.4%) patients were from Zelazle *Kebelle* (AR = 13.6/1000) and the rest from Tsehayo (AR = 10.3/1000) (Fig. 3). Of the total, 126 (86.9%) infections were caused by *Plasmodium falciparum* and the rest by *Plasmodium vivax*.

### Analytic results

#### Socio demographic characteristics

In the case-control study, a total of 60 cases and 120 controls were interviewed with a response rate of 96.8%. Cases and controls differed by sex (71.7% vs 49.2% male, *p* = 0.005) and age (68.3% vs 96.6% aged > 15 years, *p* = 0.000 (Table 2). The most common symptoms among cases-patients were fever (93.3%), headache (73.3%), and anorexia (70.0%).

**Table 2:** Socio demographic characteristics of respondents of malaria outbreak in Laelay Adyabo district northern Ethiopia, 2017

Variables	Categories	Cases (*n* = 60)	Controls (*n* = 120)
		No (%)	No (%)
Age range	< 5 years	5 (8.3)	0
	5–14 years	14 (23.3)	4 (3.3)
	≥15 years	41 (68.3)	116 (96.6)
Sex	Male	43 (71.7)	59 (49.2)
	Female	17 (28.3)	61 (50.8)
Occupation	Daily laborer	7 (11.7)	11 (9.2)
	Student	13 (21.7)	4 (3.3)
	Farmer	31 (51.7)	75 (62.5)
	Others^a^	9 (15.1)	30 (25)
Marital status	Married	22 (36.6)	91 (75)
	Single	36 (60)	20 (16.5)
	Others^b^	2 (3.4)	9 (7.5)
Educational status	No formal education	24 (40)	57 (47.5)
	Primary (1-8th)	33 (55)	54 (47.5)
	Secondary +	3 (5)	9 (7.5)

^a^underage, government employer, merchant ^b^widowed and divorced

Fourteen (23.3%) case-patients were treated within 24 h of onset while the rest were treated at least 1 day after onset. Forty-eight (80%) case-patients were infected with *Plasmodium falciparum* and 12 (20%) by *Plasmodium vivax* and they were treated by coartem and chloroquine, respectively.

#### Risk factors for malaria

Multiple factors were associated with illness in bivariate analysis. Variables with *p* < 0.2 were entered into multivariable analyses (Table 3). In multivariable analysis, cases had 73% lower odds (AOR = 0.27, 95% CI 0.11–0.66) of using protective clothing during the night compared with controls. Cases had 75% lower odds (AOR = 0.25 95% CI 0.11–0.61) of having waste collection material in their house than controls, and 75% lower odds (AOR = 0.25 95% CI 0.08–0.75) of knowing the mode of malaria transmission than controls. Cases also had nine-fold higher odds (AOR = 9.08 95% CI 3.6–22.93) of having mosquito breeding sites around their homes than controls, and nearly four times higher odds (AOR = 3.7 95% CI 1.44–9.56) of staying outdoors overnight than controls (Table 3).

**Table 3:** Factors associated with malaria outbreak in Laelay Adyabo district, Northern Ethiopia 2017

Factors	Category	Cases *n* = 60 No (%)	Controls *n* = 120 No (%)	COR (95% CI)	AOR (95% CI)
Sex	Male	43 (71.7)	59 (49.2)	2.62 (1.34–5.09)*	0.89 (0.33–2.36)
	Female	17 (28.3)	61 (50.8)	1.00	
Outdoor over night	Yes	28 (46.7)	17 (14.2)	4.32 (2.16–8.67)*	3.7 (1.44–9.56)**
	No	32 (53.3)	103 (85.8)	1.00	
ITNs	Yes	46 (76.7)	102 (85)	0.58 (0.27–1.27)	0.97 (0.31–3.01)
	No	14 (23.3)	18 (15)	1.00	
Mosquito breeding site	Yes	49 (81.7)	34 (28.3)	11.27 (5.24–24.21)*	9.08 (3.6–22.93)**
	No	11 (18.30	86 (71.7)	1.00	
Repellent	Yes	02 (3.3)	24 (20)	0.14 (0.03–0.61)*	0.35 (0.06–1.92)
	No	58 (96.7)	96 (80)	1.00	
Protective cloths	Yes	20 (33.3)	82 (68.3)	0.23 (0.12–0.45)*	0.27 (0.11–0.66)**
	No	40 (66.7)	38 (31.7)	1.00	
Waste collection material in their house	Yes	15 (25)	86 (71.7)	0.13 (0.06–0.27)*	0.25 (0.11–0.61)**
	No	45 (75)	34 (28.3)	1.00	
Intermittent river	Yes	39 (65)	19 (15.8)	9.87 (4.8–20.33)*	2.13 (0.8–5.65)
	No	21 (35)	101 (84.2)	1.00	
Knowledge on Sign and symptom	Good	52 (86.7)	115 (95.8)	0.28 (0.09–0.91)*	0.99 (0.14–7.18)
	Poor	8 (13.3)	5 (4.2)	1.00	
Knowledge on transmissions	Good	42 (70)	107 (89.2)	0.28 (0.13–0.63)*	0.25 (0.08–0.75)**
	Poor	18 (30)	13 (10.8)	1.00	

*Significant variable in bivariate analysis ** significant variables in multivariable analysis

#### Environmental observations

In all observed households, we noted unnecessary weeds around the house, artificial water holding containers, especially broken gutters, unused cans, unused old ties, and availability of un-cleaned dungs. Large traditional gold mining site was available separated from the community. There were also stagnant waters especially at Zelazle *Kebelle*.

#### Public health intervention

The affected *Kebelles* were assessed by health center rapid response teams and district public health emergency management focal personnel, and reviewed for risk factors. Most of the mosquito breeding sites were removed and indoor residual spray of Bendiocarb chemical was sprayed in all household in both *Kebelles*. Health education was given to the community of the district on comprehensive knowledge of malaria transmission and prevention modalities. Temporary sites for diagnosis and treatment were opened to interrupt further transmission by early treatment.

## Discussion

Malaria outbreak was investigated in Laelay Adyabo district northern Ethiopia. The overall attack rate was 12.1 cases per 1000 population, this finding was lower than a study done in Asgede Tsimbla district, Ethiopia (attack rate: 22.3) and India (attack rate: 15.1) [14, 21] but greater than a study done in Pakistan (attack rate: 0.9) [22]. The low attack rate in this study might be attributed to area difference in the burden of malaria and duration of the illness. This area is known as low transmission area for malaria and the duration of illness was short compared to other studies [7, 23, 24].

Median age of malaria patients were 16 years, which is in line with outbreak investigation done in Ankesha and Asgede Tsimbla districts, Ethiopia [10, 14], but younger than the study done in Guna Yala, Panama where the median age was 25 years [25]. This might be due to majority of the adolescents were spending more time outdoors in this area for agricultural and livestock keeping activities [6].

Most of the patients (72%) were males, this is also similar with the studies done in Ethiopia (Ziway and Amhara region pilot study), but higher than a study done in India (50.6%) and Ethiopia (Asgede Tsimbla (58%) and Bunga (69%)) [6, 13, 14, 23]. This could be due to the reason that males were more likely engaged in agricultural, livestock keeping and traditional gold mining activities than females in our study area than the other studies. Most of male adolescent population in those *Kebelles* engaged on traditional gold mining, most of the summer period stays outdoor overnight and also during the study period there was interruption of rain fall. This implies that the regional health bureau needs to give more focus and extend medical services to people who are engaged in agriculture, livestock keeping and traditional gold mining.

In this study most of the cases were caused by *Plasmodium falciparum* (86.9%). This is similar with the previous year report of the district and by Tigray regional health bureau report. This result also similar with the study done in Asgede Tsimbla and Setit Humera, Ethiopia [3, 7, 13, 14]. As the species of *Plasmodium* were identified by microscope and rapid diagnostic test the high amount of *Plasmodium falciparum* could be due to the areas which are in the same region.

Peoples who wore protective cloth at night, knowing about malaria mode of transmission, and availability of waste collection material in their house were less likely to be diseased than those who didn’t wore protective cloth at night, less knowledgeable on mode of transmission, and have waste collection material in their houses. This is consistent with the study done in Zimbabwe [11]. But it is different from the studies conducted in Ameya and Asgede Tsimbla districts of Ethiopia [9, 14]. This may be due to the difference in study period and malaria intervention measures of the study areas which is in our area the health personnel’s were educated the community about malaria mode of transmission and prevention modalities [3, 4, 7, 9, 13, 14]. These findings indicate that comprehensive awareness creation measures on prevention and transmission modalities of malaria are required to mitigate the outbreak effectively.

Those who had mosquito breeding site around their houses were more likely to be diseased by malaria than those who didn’t have mosquito breeding site. This is similar with study done in Ankesha, Ameya, Setit Humera, and Asgede Tsimbla districts of Ethiopia [9, 10, 13, 14]. This could be due to the presence of mosquito breeding site that created suitable condition for reproduction of mosquito and infecting more people since most of the populations stayed out door during the night. The other reason might be due to availability of more stagnant waters and other suitable factors for mosquito breeding sites due to an interruption of rainfall during the study period. Also it could be due to the fact that most of the populations were engaged in outdoor activities especially during the study period. To control malaria outbreak, mosquito breeding sites need to be cleared by involvement of the local community.

In the study those who developed malaria were three times staying out door overnight than those who didn’t develop malaria. This is consistent with the study done in India, Setit Humera, and Asgede Tsimbla districts of Ethiopia [13–15]. This may be due to the fact that mosquitos were abundant during night and most of the peoples were not using any protective modalities during night and less use of ITNs outside their home. This indicated that the local health office should educate people staying out-door to use protective modalities.

Limitations of the study include that health facilities registration book didn’t record all relevant variables and that there were no malaria data during the past 5 years which was the best to set baseline levels of malaria cases; thus, the assessment of an outbreak was done based on doubling the previous year in similar weeks.

### Conclusions and recommendation

The overall attack rate for malaria during this outbreak was high affecting > 1% of the population. This malaria outbreak was mostly affected for those male populations and residence in Zelazle *Kebelle*. Even though there are efforts to eliminate malaria in low transmission areas, the factors responsible to maintain malaria were not well controlled. Factors including presence of mosquito breeding sites around the home, staying outdoor overnight, wearing protective close during night, good knowledge on malaria mode of transmission, and presence of waste collection material at home are predictors of the infection.

Therefore, further creation of awareness to the community on malaria mode of transmission and prevention modalities and empowerment of community on their environmental cleanness may mitigate malaria outbreak. The local health workers and decision makers better to keep clean environment by empowering the community themselves and also better to create prevention modalities like preventive cloths and another insecticidal repellents to those outdoor at the night population and to people of special need.

## Additional file

Additional file 1: Questionnaire for risk factors of malaria outbreak in Laelay Adyabo district Northern Ethiopia, 2017. (DOCX 18 kb)

## Abbreviations

AOR: Adjusted Odds Ratio
AR: Attack Rate
CI: Confidence Interval
COR: Crude Odds Ratio
IRS: Indoor Residual Spray
ITNs: Insecticidal Treated Net
RDT: Rapid Diagnostic Test
VIF: Variance Inflation Factor
WHO: World Health Organization

## References

[1] WHO (2016). Malaria fact sheet.

[2] WHO (2015). World malaria report.

[3] FMOH (2015). Health and health related indicator.

[4] FMOH (2012). National malaria guideline.

[5] EPHTI (2006). Internal medicine lecture note, malaria.

[6] Seyoum A, Balcha F, Balkew M, Ali A, Gebre-Michael T (2002). Impact of cattle keeping on human biting rate of anopheline mosquitoes and malaria transmission around Ziway. Ethiopia East Afr Med J.

[7] Laelay adyabo district health office. Annual report of public health emergency management, malaria report: Adi Daero; 2017. p. 12–8.

[8] Tamiru MA, Kassa AW, Beyene BB, Mossie TB, Mekonnen YA (2014). Malaria outbreak investigation in Mecha, Dera and Fogera districts, Amhara region, Ethiopia. Am J Health Res.

[9] Defi GB, Belachew A, Addissie A, Hailemariam Z (2015). A Malaria outbreak in Ameya Woreda, south-west Shoa, Oromia, Ethiopia, 2012: weaknesses in disease control, important risk factors. Am J Health Res.

[10] Lake MW, Mebratu M, Mehari D, Dessie K (2016). Epidemiological analysis of Malaria outbreak in Ankesha District, Awi zone, Amhara region, Ethiopia, 2012: weaknesses in control measures and risk factors. Sci J Public Health.

[11] Mugwagwa N, Mberikunashe J, Gombe NT, Tshimanga M, Bangure D, Mungati M (2015). Factors associated with malaria infection in Honde valley, Mutasa district, Zimbabwe. BMC Res Notes J.

[12] Lautze J, McCartney M, Kirshen P, Olana D, Jayasinghe G, Spielman A (2007). Effect of a large dam on malaria risk: the Koka reservoir in Ethiopia. J Trop Med Int Health.

[13] Gidena D (2014). Malaria outbreak investigation in Setit Humera town.

[14] Gebrezgiabher E (2016). Malaria outbreak investigetion in asgede tsimbla district.

[15] Sharma PK, Ramanchandran R, Hutin YJ, Sharma R (2009). Gupte MD. A malaria outbreak in naxalbari, Darjeeling district West Bengal India, 2005: weeknes in disease control and risk factors. Malar J.

[16] USAID, CDC (2015). Malaria operational plan fasical tear, 2019.

[17] FMOH (2015). Malaria symposium and world malaria day; Proceedings of the annual review meeting.

[18] Yukich JO, Taylor C, Eisele TP, Reithinger R, Nauhassenay H, Berhane Y (2013). Travel history and malaria infection risk in a low-transmission setting in Ethiopia: a case control study. Malar J.

[19] Cotter C, Sturrock H, Hsiang M, Liu J, Phillips A, Hwang J (2013). The changing epidemiology of malaria elimination: new strategies for new challenges. Lancet J.

[20] EPHI (2016). Malaria Indicator Survey.

[21] Sharma R. Rashmi Sharma, Epidemiological investigation of malaria outbreak in village Santej, district Gandhi Nagar (Gujarat). Indian Journal Preventive Social Medicine. 2006;37(3-4):125-132.

[22] Prakash A, Bhattacharyya DR, Biswas D, Mohapatna PK. Outbreak investigation of malaria in North Lakhimpur district,Pakistan. Pakistan: 2007; 47-51.

[23] Calada J, Ricara M, Chystrie R, Carlos V, Manuel D, Luis FC etal. Characterization of a recent malaria outbreak in the autonomous indigenous region of Guna Yala, Panama, Malaria Journal, 2015;14(459): 1-10. 10.1186/s12936-015-0987-6. 10.1186/s12936-015-0987-6 PMC4650261 26578076

[24] Yeshiwondim AK, Scott C, Guinovart C, Serda B, Tesfay BH, Agmas A, et al. Case Investigation With Reactive Focal Testing and Treatment For Malaria in a Low-transmission Area In Amhara, Ethiopia, malaria journal, 2015; 17(449): 1-11. 10.1186/s12936-018-2587-8 PMC6278130 30514307

[25] Lee P-W, Liu C-T, Rosario VE, Sousa Bd, Rampao HS, and Shaio M-F. Potential threat of malaria epidemics in a low transmission area, as exemplified by São Tomé and Príncipe, malaria journal; 2010, 9(264):1-12. 10.1186/1475-2875-9-264 PMC2955676 20920216

