# Supplementary material for: Risk factors associated with malaria outbreak in Laelay Adyabo district northern Ethiopia, 2017: case-control study design
Source: BMC Public Health. 2019 May 2;19:484. doi: 10.1186/s12889-019-6798-x (PMC6498533; doi:10.1186/s12889-019-6798-x)
Supplement: Supplementary file 1 — Questionnaire for risk factors of malaria outbreak in Laelay Adyabo district Northern Ethiopia, 2017. (DOCX 18 kb) [file 12889_2019_6798_MOESM1_ESM.docx]

**Informed consent sheet**

Mekelle University, College of Health Sciences, School of Public Health, Epidemiology department

Hello! My name is........................................................... And I am working in ____________ health center and I am collecting data on the study conducted risk factors of malaria outbreak in Laelay Adyabo district Tigray Ethiopia 2017. I would like to ask you about your aspects on factors associated to malaria. This information will help the district, region and country as well as other stakeholder to plan malaria prevention and control mechanisms. The questionnaire only takes between 15 and 20 minutes to complete. The information you provide will be kept strictly confidential and will not be shown to other persons and your participation is definitely important to identify the risk factors of malaria outbreak. There are no any incentives or direct benefits as well as risks in participating in this outbreak investigation project. Participation in this study is voluntary and you can choose not to answer any individual question or all of the questions. However, I hope that you will participate in this study since your views are important.

Consent (Verbal and written Consent):-

Respondent agrees to be interviewed Start interview.............. 1

Respondent doesn't agree to be interviewed Terminate Interview........... 2

Name of interviewer: __________________________ Signature ____________

Date: ______________

Data collector mobile number ___________________________________________

**1. Socio-demographic information:**

1.1. ID number of respondent______

1.2. Age in years_____

1.3. Sex: M__________F____

1.4. Address: Region _______Zone_________ District ________ kebelle __________ village_________

1.5. Occupation: 1) gov’t employed 2) daily laborer 3) student 4) farmer 5) Merchant 6) other_________

1.6. Total family members’ ___________

1.7. Ethnicity: _____________

1.8. Religion: 1) orthodox 2) protestant 3) Muslim 4) other ___________

1.9. Marital status: 1) Married 2) single 3) Widowed 4) Divorced

1.10. Educational status:

1) Unable to write and read 2) able to write and read without formal education

3) Primary (1-8^th^) 4) secondary (9^th^-12^th^) 5) tertiary (>12 year)

**2. Clinical presentations:**

*(For case only)

2.1. What was the first symptom? _____

2.2. When was the 1^st^ symptom started (date of onset of symptoms) DD/MM/YY_________________

2.3. What were symptoms?

a) Fever: yes____no____

If yes duration of fever ______was it constant fever? Yes ___ no_____every other day_______

b) Vomiting

d) Anorexia

e) Head ache

f) Sweating

g) Chilling and shivering

h) Weakness

i) Cough j) Back pain

k) Muscle pain

l) Rigor

2.4. Result ________________________

2.5. Did you get any treatment? Yes__ no ___if yes, what treatment did you get?

a) Coartem b) Chloroquine

c) Quinine tablets

d) Quinine injection (e) Other ____________

**3. Risk Factors: (For both cases and controls)**

3.1. Sleeping areas in side home ___________outside home_________

3.2. Did you stay outside overnight (after 6:00 pm)? Yes___ no ____

3.3. Did you travel outside your village in the past 2-3 weeks: - yes­­­____ no___

3.4. Is there a similar sick patient in your household yes----no----

3.5. If yes Q 3.4, the number of sick individuals ______________________

3.6. Do you have bed net in your household yes ___no ___

3.7. If yes Q 3.6, how often do you use

A) Always b) sometime c) never

3.8. If yes Q 3.6 the number of bed nets ______

3.9. If yes Q 3.6 Do mothers and children given priority of using bed nets? Yes __no ___

3.10. Was indoor residual sprayed (IRS) this year? Yes___no___

3.11. If yes Q3.10 when?(DD/MM/YY _______________________

3.12. If yes to Q3.10 how often? Once___twice___ three times______

3.13. History of malaria in the past two months: - yes no

3.14. If yes Q3.13, did you treated for the malaria? Yes no

**4. Environmental investigation**

4.3. Presence of mosquito vectors/ mosquitoes breeding sites around the home or vicinity?

a) Yes b) no

4.4. If Q 4.3 yes, presence of larvae in breeding site: - a) yes b) no

4.5. Type of house: a) mad b) cement c) stone d) others_________________

4.6. Do you use repellents: - a) yes b) no

4.7. Protective clothing at night (long close cover hands and legs): - a) yes b) no

4.8. Waste collection: a) yes b) no

4.9. Unprotected irrigation: - a) yes b) no

4.10. Presence of intermittent rivers close to the community: - a) yes b) no

**5. Awareness assessment**

5.1. What are the sign and symptom of malaria (possible to answer more than one answer)

Yes No

1.Fever 1 0

2.Vomiting 1 0

3.Anorexia 1 0

4.Chills & shiver 1 0

5.Headache 1 0

6.Sweating 1 0

7.Back pain 1 0

8.Arthralgia 1 0

9.Weakness 1 0

5.2. How it transmitted?

Yes no

1.By mosquito bite 1 0

2.Blood transfusion 1 0

3.Mother to child 1 0

4.By flies 1 0

5.Breathing 1 0

6.Body contact 1 0

7.By hunger 1 0

5.3. How it can be prevented?

Yes no

1.Early diagnosis &treatment 1 0

2.House spray by insecticidal 1 0

3.Use of mosquito bed net 1 0

4.Environmental hygiene 1 0

5.By using good nutrition 1 0
